# The Role of T cell PPAR γ in mice with experimental inflammatory bowel disease

**DOI:** 10.1186/1471-230X-10-60

**Published:** 2010-06-10

**Authors:** Amir J Guri, Saroj K Mohapatra, William T Horne, Raquel Hontecillas, Josep Bassaganya-Riera

**Affiliations:** 1 Nutritional Immunology and Molecular Nutrition Laboratory, Virginia Bioinformatics Institute, Virginia Polytechnic Institute and State University, Blacksburg, VA 24061, USA

## Abstract

**Background:**

Peroxisome proliferator-activated receptor γ (PPAR γ) is a nuclear receptor whose activation has been shown to modulate macrophage and T cell-mediated inflammation. The objective of this study was to investigate the mechanisms by which the deletion of PPAR γ in T cells modulates immune cell distribution and colonic gene expression and the severity of experimental IBD.

**Methods:**

PPAR γ flfl; CD4 Cre^+ ^(CD4cre) or Cre- (WT) mice were challenged with 2.5% dextran sodium sulfate in their drinking water for 0, 2, or 7 days. Mice were scored on disease severity both clinically and histopathologically. Flow cytometry was used to assess lymphocyte and macrophage populations in the blood, spleen, and mesenteric lymph nodes (MLN). Global gene expression in colonic mucosa was profiled using Affymetrix microarrays.

**Results:**

The deficiency of PPAR γ in T cells accelerated the onset of disease and body weight loss. Examination of colon histopathology revealed significantly greater epithelial erosion, leukocyte infiltration, and mucosal thickening in the CD4cre mice on day 7. CD4cre mice had more CD8^+ ^T cells than WT mice and fewer CD4^+^FoxP3^+ ^regulatory T cells (Treg) and IL10^+^CD4^+ ^T cells in blood and MLN, respectively. Transcriptomic profiling revealed around 3000 genes being transcriptionally altered as a result of DSS challenge in CD4cre mice. These included up-regulated mRNA expression of adhesion molecules, proinflammatory cytokines interleukin-6 (IL-6) and IL-1β, and suppressor of cytokine signaling 3 (SOCS-3) on day 7. Gene set enrichment analysis (GSEA) showed that the ribosome and Krebs cycle pathways were downregulated while the apoptosis pathway was upregulated in colons of mice lacking PPAR γ in T cells.

**Conclusions:**

The expression of PPAR γ in T cells is involved in preventing gut inflammation by regulating colonic expression of adhesion molecules and inflammatory mediators at later stages of disease while favoring the recruitment of Treg to the mucosal inductive sites.

## Background

Inflammatory bowel disease (IBD), with its two clinical manifestations Crohn's Disease (CD) and Ulcerative Colitis (UC), is a widespread and debilitating disease characterized by inflammation and immune cell infiltration and immune-mediated destruction of the gastrointestinal tract [1]. Activation of the nuclear receptor peroxisome proliferator-activated receptor γ (PPAR γ) has demonstrated efficacy in reducing the severity of IBD by suppressing excessive immunoinflammatory responses [2-4]. In the gut, PPAR γ is highly expressed in epithelial cells, macrophages, and T-cells [5], and its activation has been shown to repress nuclear factor-κB (NF-κB)-mediated inflammation and promote a regulatory, anti-inflammatory phenotype [2,6,7].

Our laboratory has shown that the deficiency of PPAR γ in hematopoietic and epithelial cells significantly impairs the ability of a naturally occurring PPAR γ ligand, conjugated linoleic acid, to improve inflammatory bowel disease [2,8,9], or inflammation-driven colorectal cancer [10]. However, it remains unclear how these effects are mediated through macrophages, T cells, epithelial cells, or a combination of cells in the intestinal lamina propria, mesenteric lymph nodes (MLN) and circulating lymphocytes. The role of PPAR γ in epithelial cells was examined by Adachi et al, who found that the deficiency of PPAR γ resulted in significantly worsened disease activity and enhanced levels of pro-inflammatory cytokines interleukin 6 (IL-6) and IL-1β following DSS-induced colitis [11]. Mohapatra et al also demonstrated that mice lacking PPAR γ in intestinal epithelial cells show upregulated expression of genes in the lysosomal pathway [12]. Shah and others demonstrated that the deficiency PPAR γ in macrophages also worsens DSS colitis [13]. However, the importance of T cell PPAR γ in the pathogenesis of IBD is less understood.

The DSS colitis model targets initially epithelial cells and macrophages, but there is a definite T cell involvement at later stages of disease [14]. Regulatory T cell (Treg) PPARγ is involved in maintaining homeostasis at the gastrointestinal tract [15,16] and preventing chronic CD4^+ ^T cell-induced colitis [17]. Thus, Treg represent an important target of endogenous and exogenous PPAR γ ligands. The objective of this study was to perform a comprehensive time course analysis of the effect of T cell-specific deletion of PPAR γ on the development of experimental IBD by using a systems approach aimed at examining immune cell distribution, global colonic gene expression and gut immunopathology.

## Methods

### Animal Procedures

PPAR γ flfl; CD4Cre^+ ^(CD4cre, n = 34) and Cre-wild-type mice (WT, n = 34) in C57BL/6J background were used for these experiments. The CD4cre mice (kindly provided by Dr. R.B. Clark, University of Connecticut) express a transgenic recombinase under the control of the CD4-Cre promoter [18]. Since all T cell precursors express the CD4 co-receptor at the thymic level (during the double-positive thymocyte stage), the PPAR γ gene is deleted from both CD4^+ ^and CD8^+ ^T cells. The mice were housed at the animal facilities at Virginia Polytechnic Institute and State University in a room maintained at 75°F, with a 12:12 h light-dark cycle starting from 6:00 AM. All experimental procedures were approved by the Institutional Animal Care and Use Committee of Virginia Polytechnic Institute and State University and met or exceeded requirements of the Public Health Service/National Institutes of Health and the Animal Welfare Act. Mice were challenged with 2.5% dextran sodium sulfate (DSS), 36,000-44,000 molecular weight (ICN Biomedicals, Aurora, OH) in the drinking water. After the DSS challenge mice were weighed on a daily basis and examined for clinical signs of disease associated with colitis (i.e., perianal soiling, rectal bleeding, diarrhea, and piloerection). For the DSS challenge, the disease activity indices and rectal bleeding scores were calculated using a modification of a previously published compounded clinical score [2]. Briefly, disease activity index consisted of a scoring for diarrhea and lethargy (0-3), whereas rectal bleeding consisted of a visual observation of blood in feces and the perianal area (0-4). Mice in the DSS study were euthanized on days 0, 2, and 7 of the DSS challenge by carbon dioxide narcosis followed by secondary thoracotomy and blood was withdrawn from the heart. Spleen and mesenteric lymph nodes (MLN) were scored based on size and macroscopic inflammatory lesions (0-3), excised, and single-cell suspensions were prepared as previously described [17] for flow cytometry.

### Histopathology

Colonic sections were fixed in 10% buffered neutral formalin, later embedded in paraffin, and then sectioned (5 μm) and stained with hematoxylin and eosin (H&E) for examination of microscopic lesions and changes in the mucosal architecture. Colons were graded with a compounded histologic score including the extent of (1) leukocyte infiltration, (2) mucosal thickening, and (3) epithelial cell erosion. The sections were graded with a score of 0-4 for each of the previous categories and data were analyzed as a normalized compounded score. Five fields per tissue and 11 mice per time point were examined.

### Immunophenotyping of blood, spleen, and mesenteric lymph nodes

MLN and spleen-derived cells or whole blood were seeded onto 96-well plates, centrifuged at 4°C at 3000 rpm for 4 minutes, and washed with PBS containing 5% serum and 0.09% sodium azide (FACS buffer). To assess differential monocyte/macrophage subsets, the cells were then incubated in the dark at 4°C for 20 minutes in FcBlock (20 μg/ml; BD Pharmingen), and then for an additional 20 minutes with fluorochrome-conjugated primary antibodies anti-F4/80-PE-Cy5 (5 μg/mL, ebioscience) and anti-CD11b-Alexa Fluor 700 (2 μg/mL, eBioscience). For lymphocyte subset assessment, cells were incubated with anti-CD4-Alexa Fluor 700 (2 μg/mL; BD Pharmingen), anti-CD8-PerCp-Cy5.5 (2 μg/mL, eBioscience), CD3 PE-Cy5 (2 μg/mL; BD Pharmingen), anti-FoxP3-PE (2 μg/mL, eBioscience), and anti-IL10-PE as previously shown [19]. Flow results were computed with a BD LSR II flow cytometer and data analyses was performed with FACS Diva software (BD).

### Microarray data analysis

After homogenization of tissue, total RNA was extracted and purified using the RNAeasy system according to manufacturer's instructions (Qiagen Valencia, CA). The QIAGEN RNase-free DNase supplement kit was used to ensure that the RNA was free from DNA contamination. RNA was then processed and labeled according to the standard target labeling protocols and the samples were hybridized, stained, and scanned per standard Affymetrix protocols at VBI core laboratory on Mouse 430 2.0 expression arrays (Affymetrix Inc., Santa Clara, CA). All statistical analysis of the data was performed within R statistical environment - Version 2.9.0 [20] using Bioconductor packages [21]. Raw microarray data from CEL files were read with 'affy' package [22] and pre-processed by gcRMA algorithm (GC Robust Multiarray Average) that performs the three steps: (i) adjustment of the gene expression signal against the background caused by optical noise and non-specific binding, (ii) robust multi-array normalization [23], and (iii) summarization of the probes belonging to each probe set. The microarray data (both raw and normalized) have been submitted at the Gene Expression Omnibus. (GEO, http://www.ncbi.nlm.nih.gov/geo/, Data set: GSE20523).

### Gene Set Enrichment Analysis

All pathways listed at Kyoto Encyclopedia for Genes and Genomes (Kyoto Encyclopedia of Genes and Genomes) [24] were selected for analysis. Two approaches were taken to find the KEGG pathways significantly affected by DSS in CD4cre mice. In the first approach, a global search was conducted to find the KEGG pathway most significantly affected under the condition. The degree of differential regulation of a pathway in CD4cre (compared to wild-type) mice after 7 days of DSS treatment was derived from a collective differential regulation of the member genes in that pathway, as described earlier [25]. Briefly, each KEGG pathway was assigned a score depending on its degree and direction of modulation, which represented changes in collective gene expression across most genes in each pathway. Normal-normal plot (QQ-plot) and permutation testing were conducted to find the pathway with a value significantly different from the rest of the pathways. In the second approach, the genes differentially expressed on day 7 of DSS treatment, as derived from pair-wise gene-level comparison between day 7 post-DSS and no DSS in CD4cre mice, were selected for exploring which KEGG pathways were over-represented in this collection of genes. These included both up-and down-regulated genes due to DSS. None of these genes were differentially expressed in the wild-type mice at corresponding time point (i.e., day 7) of DSS treatment. Hypergeometric testing was performed on this collection of genes to discover the over-represented KEGG pathways. This procedure used Fisher's exact test to find association between interesting genes (differentially expressed at day 7 in CD4cre mice) and membership to a KEGG pathway. Results from both approaches were combined to list the KEGG pathways that are significantly modulated by DSS on day 7 in CD4cre mice.

### KEGG pathway visualization

KEGG pathways found to be significantly associated with DSS on day 7 in CD4cre mice were accessed using the bioconductor package KEGGSOAP. Specific gene nodes on each pathway were 'colored' according to the direction of differential expression of that gene in CD4cre mice on day 7 of DSS treatment: red if up-regulated, green if down-regulated.

### Quantitative Real-Time RT-PCR

To validate microarray results we used quantitative, real-time, RT-PCR. Total RNA (1 μg) from colons was used to generate a complementary DNA (cDNA) template using the iScript cDNA Synthesis Kit (Bio-Rad, Hercules, CA) using previously described conditions [2]. Each gene amplicon was purified with the MiniElute PCR Purification Kit (Qiagen) and quantitated on an agarose gel by using a DNA mass ladder (Promega). These purified amplicons were used to optimize real-time PCR conditions and to generate standard curves in the real-time PCR assay. Primer concentrations and annealing temperatures were optimized for the iCycler iQ system (Bio-Rad) for each set of primers using the system's gradient protocol. PCR efficiencies were maintained between 92 and 105% and correlation coefficients above 0.98 for each primer set during optimization and also during the real-time PCR of sample DNA.

Complementary DNA (cDNA) concentrations for genes of interest were examined by real-time quantitative PCR using an iCycler IQ System and the iQ SYBR green supermix (Bio-Rad). A standard curve was generated for each gene using 10-fold dilutions of purified amplicons starting at 5 pg of cDNA and used later to calculate the starting amount of target cDNA in the unknown samples. SYBR green I is a general double-stranded DNA intercalating dye and may therefore detect nonspecific products and primer/dimers in addition to the amplicon of interest. In order to determine the number of products synthesized during the real-time PCR, a melting curve analysis was performed on each product. Real-time PCR was used to measure the starting amount of nucleic acid of each unknown sample of cDNA on the same 96-well plate.

### Statistics

Flow cytometry, disease activity, pathology and real-time RT-PCR data were analyzed as a completely randomized design. To determine the statistical significance of the model, analysis of variance (ANOVA) was performed using the general linear model procedure of Statistical Analysis Software (SAS), and probability value (*P*) < 0.05 was considered to be significant. When the model was significant, ANOVA was followed by Fisher's Protected Least Significant Difference multiple comparison method. Statistical analysis for microarray analyses was performed using the software package "limma" [26], for pairwise comparisons (Day 2 post-DSS Vs. no DSS, Day 7 post-DSS Vs. no DSS). P-values for the comparisons of interest were obtained using empirical Bayes method [27] and adjusting for multiple testing [28]. Venn Diagrams were drawn to show the number of genes differentially expressed due to DSS challenge at different time points (days 2 or 7), in different mouse strains (WT or CD4cre) and the genes commonly induced in both strains. A large number of genes found to be differentially expressed on day 7 of DSS challenge in CD4cre mice were further subjected to hypergeometric testing [29] for identifying the enriched KEGG [30] pathways as described in the Gene Set Enrichment Analysis section.

## Results

### The loss of PPAR γ in T cells accelerates the onset of disease activity and body weight loss

**Figure 1:**
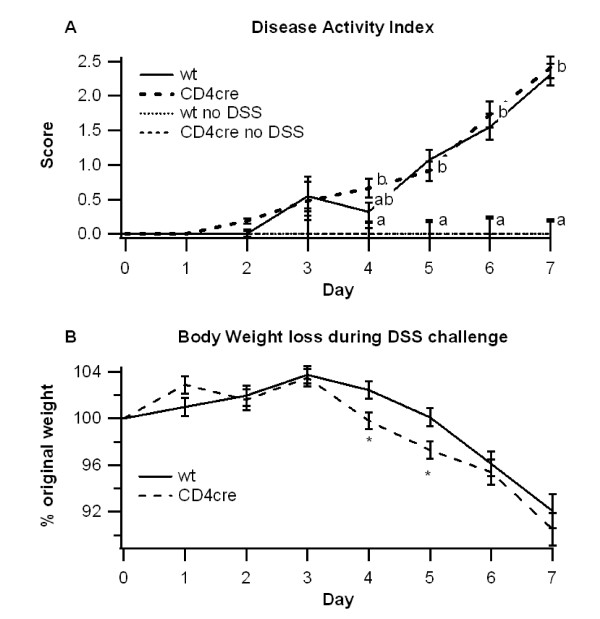
**Effect of T cell-specific PPAR γ deletion on disease severity**. WT or CD4cre mice were treated with 2.5% dextran sodium sulfate (DSS) or water (no DSS) for 7 days. The disease activity index (DAI), a composite score reflecting clinical signs of the disease (i.e. perianal soiling, rectal bleeding, diarrhea, and piloerection) was assessed daily (A) and the average daily loss in body weights (B) throughout the 7 day DSS challenge was calculated. Data are represented as mean ± standard error. Points with different subscripts are significantly different (*P *< 0.05).

WT or CD4cre mice were challenged with 2.5% DSS for 7 days to induce experimental IBD and to determine the effect of T cell-specific deletion of PPAR γ on colitis severity, gene expression and immune cell distribution. Differences between the groups first appeared on day 4 of the challenge, where CD4cre mice had significantly greater body weight loss despite no significant difference in disease activity (Figure 1A and 1B). The differences in body weights were transient however, and by day 7 body weights were relatively equal between groups.

**Figure 2:**
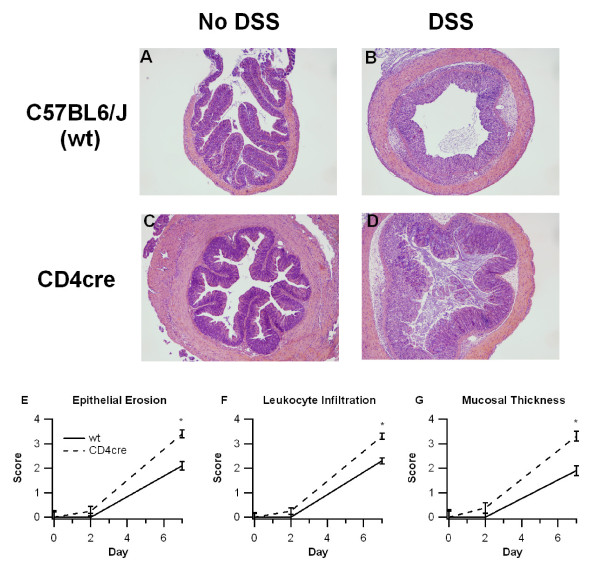
**Effect of T cell-specific PPAR γ deletion on colon histopathology**. WT or CD4cre mice were treated with 2.5% dextran sodium sulfate (DSS) or water (no DSS) for 7 days. Representative photomicrographs (40 ×) from wt, no-DSS (A), wt, DSS (B), CD4cre, no-DSS (C), and CD4cre, DSS (D) groups are depicted. All specimens underwent blinded histological examination and were scored (1-4) on epithelial erosion (E), leukocyte infiltration (F), and mucosal wall thickening (G) on days 0, 2, and 7 of DSS challenge. Data are represented as mean ± standard error. Points with an asterisk are significantly different at a given time point (*P *< 0.05).

To more closely examine the effect of T cell-specific PPAR γ deletion on experimental IBD, colons were histologically examined for the presence of inflammatory lesions and scored (Figure 2). Our analysis indicated that colons recovered from CD4cre mice had significantly more epithelial erosion, leukocyte infiltration, and mucosal thickness on day 7 compared to their WT counterparts. As anticipated from a model that initially targets epithelial cells and macrophages, no histological differences were found between WT and CD4cre mice on day 2. The leukocyte infiltration in the colon was polymorphonuclear and lymphoplasmacytic on day 2, but progressed towards a predominantly lymphoplasmacytic infiltrate on day 7 of DSS colitis.

### T cell-specific PPAR γ-deficient mice have more CD8^+ ^T cells, and fewer CD4^+ ^T cells and regulatory T cells

**Figure 3:**
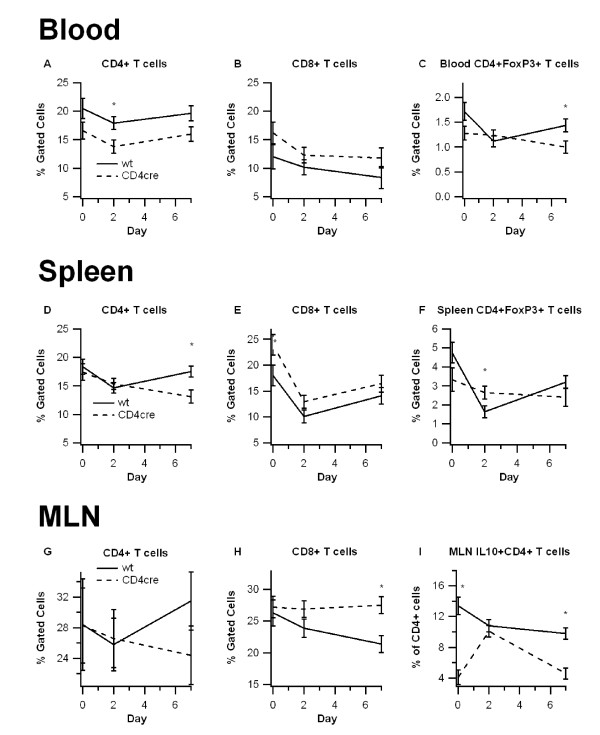
**Effect of T cell-specific PPAR γ deletion on lymphocyte subsets in blood, spleen, and mesenteric lymph nodes (MLN)**. Blood (A-C), spleen (D-E), and MLN (F-H) from wild-type (WT) or PPAR γ flfl; CD4 Cre+ (CD4cre) mice were immunophenotyped with anti-CD3, anti-CD4, anti-CD8 antibodies, anti-F4/80, anti-CD11b, anti-FoxP3, and anti-IL10 to identify immune subsets through flow cytometry. Data were collected on days 0, 2, and 7 of DSS challenge and was analyzed with FACS Diva software. Data are represented as mean ± standard error. Points with an asterisk are significantly different at a given time point (*P *< 0.05).

We next characterized the effect of T cell-specific PPAR γ deletion on the distribution of immune cell subsets in the blood, spleen, and MLN by using flow cytometry. In these tissues the CD4cre mice showed a consistent increase in the percent of CD8^+ ^T cells and a decrease in the percent of CD4^+ ^T cells (Figure 3). The number of F4/80^+^CD11b^+ ^monocytes/macrophages in these tissues was unaffected by the deletion of PPAR γ in T cells (data not shown).

We also examined the percentages of regulatory T cells and those expressing IL-10 in these tissues. On day 7, blood from WT mice had significantly more CD4^+^FoxP3^+ ^Tregs in comparison to blood from CD4cre mice. While there were no significant differences in the number of CD4^+^FoxP3^+ ^cells in MLN (data not shown), the percent of CD4^+ ^T cells expressing IL-10 was significantly lower in MLN of CD4cre mice in comparison to WT mice (Figure 3). These data suggest that the deficiency of PPAR γ in T cells impairs the Treg-mediated immunoregulation.

### Global gene expression analyses in the colonic mucosa of WT and T cell-specific PPAR γ null mice with DSS colitis

Pairwise comparisons revealed a transcriptional effect of DSS in the colonic mucosa at both time points (Day 2 and 7), with some differences between the two mouse strains. On day 2, there were more genes affected in WT (202) compared to only 8 genes in CD4cre mice (Additional file 1: Supplemental Figure S1). However, the number of genes transcriptionally altered was reduced from 200 to 39 in the WT mice on day 7. On the other hand, CD4cre mice revealed a steep rise in the number of genes differentially expressed in the later time point; from 8 on day 2 to 3036 on day 7 (Additional file 2: Supplemental Figure S2). Since CD4cre and WT mice primarily differ in the presence of T cell PPAR γ, the massive transcriptional modulation on day 7 in CD4cre mice is likely caused by functional changes in T cells derived from the deletion of PPAR γ. Out of the 3036 genes differentially expressed on day 7 in CD4cre mice, some genes were also affected under other conditions with 2990 genes being uniquely modulated on day 7 in CD4cre mice. Additionally, these 2990 genes included leukocyte extravasation markers and pro-inflammatory cytokines described and validated by real-time RT-PCR.

### Leukocyte extravasation markers and inflammatory cytokines are upregulated in colons of T cell-specific PPAR γ-deficient mice

**Figure 4:**
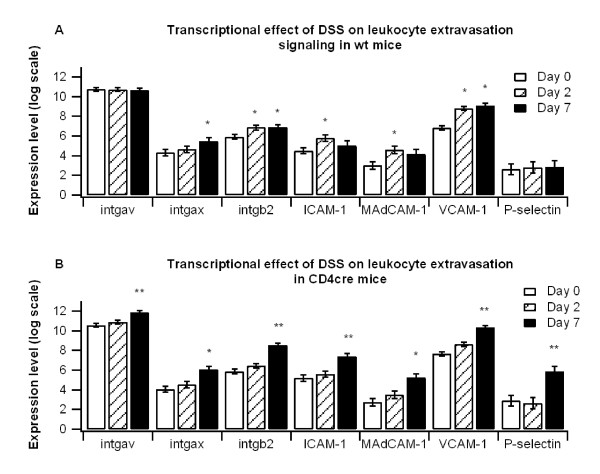
**Effect of T cell-specific PPAR γ deletion on leukocyte extravasation markers**. WT or CD4cre mice were treated with 2.5% dextran sodium sulfate (DSS) for 7 days, and microarray analyses was performed. Colons of WT (A) and CD4cre (B) mice were assessed for leukocyte extravasation markers integrin alpha V (intgav), integrin alpha X (intgax), integrin beta 2 (intgb2), intracellular adhesion molecule 1 (ICAM-1), mucosal addressin adhesion molecule 1 (MAdCAM-1), vascular adhesion molecule 1 (VCAM-1) and P-selectin. Results from microarray were validated with real time PCR. Points with an asterisk are significantly different from the day 0 time point (*P *< 0.05). Points with a double asterisk also indicate a significant difference due to genotype at a given time point (*P *< 0.05).

To determine the effect of T cell-specific PPAR γ-deletion on colonic gene expression microarrays were performed from mice on days 0, 2, and 7 of DSS challenge (Figure 4). Leukocyte extravasation markers were significantly enhanced in both WT and CD4cre mice throughout the DSS challenge, though in comparing the two groups we observed that levels were significantly greater in CD4cre mice on day 7. Expression of integrin alpha V (intgav), integrin beta 2 (intgb2), intracellular adhesion molecule (ICAM-1), vascular cell adhesion molecule (VCAM-1), and P-selectin were all significantly enhanced in CD4cre mice compared to the WT mice.

**Figure 5:**
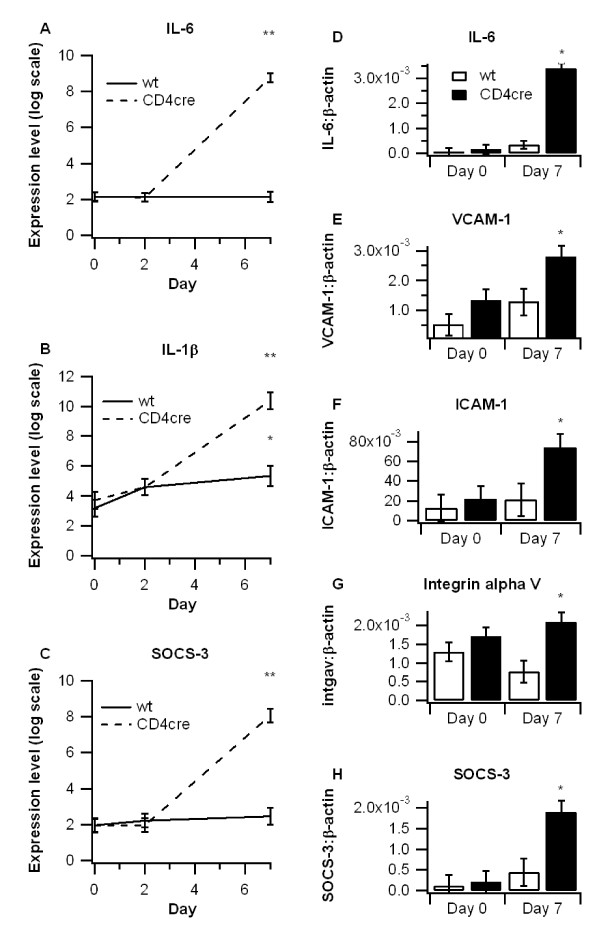
**Effect of T cell-specific PPAR γ deletion on inflammatory gene expression**. WT or CD4cre mice were treated with 2.5% dextran sodium sulfate (DSS) for 7 days, and microarray analyses was performed on colon samples. Differences in interleukin 6 (IL-6) (A), IL-1β (B), and suppressor of cytokine signaling 3 (SOCS-3) (C) were compared among groups and validated with real time PCR. Expressions of (IL-6 (D), vascular adhesion molecule 1 (VCAM-1) (E), intracellular adhesion molecule 1 (ICAM-1) (F), integrin alpha V (intgav) (G), and SOCS-3 (H) on days 0 and 7 were examined by real time PCR to validate microarray findings. Points with an asterisk are significantly different from the day 0 time point (*P *< 0.05). Points with a double asterisk also indicate a significant difference due to genotype at a given time point (*P *< 0.05).

We also examined the mRNA expression of pro-inflammatory cytokines in colons of WT and CD4cre mice. Expression of interleukin 6 (IL-6) and IL-1β were significantly higher in colons from CD4cre mice at day 7 (Figure 4). Levels of cytokine signaling 3 (SOCS-3), an inhibitory protein shown to increase in response to IL-6 that has been shown to be enhanced in both human IBD and murine models of the disease [31-33], was also significantly upregulated in colons of CD4cre mice. Microarray findings were validated with real time RT-PCR analyses (Figure 5D-H).

### The Krebs cycle and ribosome pathways are significantly downregulated in colons of T cell-specific PPAR γ null mice; while apoptosis is significantly upregulated

We next examined the effect of T cell-specific PPAR γ deletion using gene set enrichment analysis (GSEA) of KEGG pathways. The following three KEGG pathways were significantly differentially regulated as revealed by hypergeometric testing on the 2990 genes differentially expressed on day 7 of DSS treatment compared with no DSS in CD4cre mice: citrate (TCA cycle) or Krebs cycle, apoptosis and ribosomal pathways (Additional file 3: Supplemental Figure S3). Additionally, ribosomal pathway was found to be the most significantly down-regulated pathway from an unbiased global search using all genes (normal Q-Q plot and permutation testing). While many genes of the Krebs cycle and ribosomal pathway were down-regulated, those of apoptosis were upregulated (Additional files 4, 5, 6: Supplemental Figures S4-6 and Additional file 7: Supplemental Table S1). The Krebs cycle pathway is involved in the maintenance of glucose homeostasis, one of the primary roles of PPAR γ. The apoptosis pathway is critically regulated by NF-κB which is repressed by PPAR γ in IBD. However, the pathway most significantly affected by the deletion of PPAR γ was the ribosome pathway (Additional file 6: Supplemental Figure S6), which controls genes involved in protein translation and synthesis.

## Discussion

Activation of PPAR γ by exogenous administration of synthetic and naturally occurring agonists or through gene therapy has demonstrated pre-clinical efficacy in the prevention and treatment of gut inflammation [2,9,34-37]. Accordingly, PPAR γ was proposed as a therapeutic target for gastrointestinal inflammation. The results of a recent randomized, double-blind, placebo controlled study demonstrate that oral administration of rosiglitazone (Avandia), a synthetic PPAR γ ligand of the thiazolidinedione class of anti-diabetic drugs, ameliorates disease activity in human UC patients [4]. Thus, dissecting the cell specificity of PPAR γ in the prevention and treatment of gut inflammation is necessary for the rational development of placebo-controlled clinical studies aimed at investigating the efficacy and tolerability of novel PPAR γ agonists in patients with IBD.

In this regard, PPAR γ is widely expressed in all major cell types involved in the immunopathogenesis of IBD, including T cells, macrophages, dendritic cells, endothelial cells and epithelial cells. Previous studies have characterized the impact of the PPAR γ deficiency in epithelial cells in DSS colitis and demonstrated that while PPAR γ expressed in the colonic epithelium has an endogenous role in protection against colitis, rosiglitazone treatment can ameliorate experimental IBD in epithelial cell-specific PPAR γ null mice [11]. Adding to this story, our laboratory has recently shown that intestinal epithelial cell specific deletion of PPAR γ enhances macrophage MHC II expression in the MLN and lysosomal pathway gene expression in the colon without significantly affecting lymphocyte populations in the blood, spleen, and MLN [12]. A targeted disruption of PPAR γ in macrophages increased recruitment of macrophages to inflammatory foci in the colon [13]. This report investigates the role of endogenously activated T cell PPAR γ in experimental IBD.

The initial phases of DSS colitis (i.e., day 2) target the innate components of the immune response (i.e., macrophages and epithelial cells), whereas T cells are recruited at later stages of disease (days 4-7) following the initial epithelial cell damage and macrophage activation. We found that disease activity and body weights were worsened in CD4cre mice at about the fourth day of DSS challenge, indicating that disease progression is accelerated when PPAR γ is absent in T cells. Histologically, colonic inflammatory lesions were limited or null on day 2 whereas significant epithelial erosion and immune cell infiltration was observed on day 7, which was more severe in mice lacking PPAR γ in T cells. Of note, the crosstalk between myeloid cells and lymphocytes represents an important component of disease progression [38]. CD4^+ ^T cells and CD4-induced production of IL-6 are both increased in the lamina propria of DSS-treated mice [39,40], and the infiltration of CD4^+ ^T cells into the colon has been shown to correlate with gut immune-mediated pathology [40].

Transcriptomic profiling of colonic samples revealed that on day 2 of DSS colitis, corresponding to the period of activation of innate immunity, there were 202 genes affected in WT and only 8 genes in CD4cre mice. In contrast, on day 7 when both innate and acquired T cell responses contribute to gut immunopathology, we found a dramatic increase in the number of differentially expressed genes (i.e., 3036) in colons of mice lacking PPAR γ in T cells versus only 39 differentially expressed genes in colons of WT mice. Specifically, on day 7 there were significant increases in IL-6, IL-1β and SOCS3 in colons of CD4cre mice. These proinflammatory cytokines have been shown to accelerate the progression of intestinal inflammation [41-43]. The SOCS3 pathway can be induced by IL-6 and plays a down-regulatory role in the development of intestinal inflammation by inhibiting signal transducer and activator of transcription (STAT3) activation and thereby suppressing expression of inflammatory cytokines [33]. Since STAT3 is highly tyrosine phosphorylated in UC and CD patients [33], the upregulation of SOCS3 represents an anti-inflammatory feedback loop. Microarray analysis also revealed a significant increase in genes involved in leukocyte extravasation in the CD4cre mice, a finding that is in line with the increased leukocyte recruitment we observed in colons of T cell-specific PPAR γ null mice. Both VCAM-1 and ICAM-1 are adhesion molecules independently associated with IBD disease severity [44], and their upregulation is associated with increased NF-κB activity and pro-inflammatory cytokine secretion [45]. In total, these findings indicate an enhanced pro-inflammatory milieu in colons of CD4cre mice on day 7 of DSS colitis mediated by increased expression of pro-inflammatory cytokines and adhesion molecules.

GSEA of KEGG pathways demonstrated that there was a significant downregulation of gene clusters involved in the maintenance of glucose homeostasis (i.e., Krebs cycle) and protein synthesis (ribosome pathway), but up-regulation of the apoptosis pathway. Together these findings indicate a dysregulated carbohydrate metabolism, suppressed protein translation and altered programmed cell death in colons of CD4cre mice (fold-change in gene expression in Supplementary Table 2). In line with our findings, carbohydrate and amino acid metabolism have recently been shown to be reduced in colons of IBD patients [46]. Considering that one of the first discovered roles of PPAR γ was the regulation of glucose homeostasis [47], it is not surprising that the deficiency of PPAR γ in T cells could cause a down-regulation of gene clusters related to Krebs cycle. In turn, these reductions may have also resulted from a generalized increase in the severity of colonic inflammation caused by the deficiency of PPAR γ in T cells, as pro-inflammatory cytokines can inhibit Krebs cycle activity [48]. The apoptosis and protein synthesis pathways are also largely influenced by inflammation. The apoptosis pathway is critically regulated by NF-κB [49] which is thought to be repressed by PPAR γ in experimental IBD [50], and protein synthesis is decreased in the inflamed colon as a result of endoplasmic reticulum (ER) stress and subsequent activation of the unfolded protein response in mouse models of IBD [51]. Thus, the pathways affected by the deficiency of PPAR γ may be secondary to more severe colitis. ER stress is also associated with induction of apoptosis via activation of an ER resident caspase (caspase-12) [52]. Further studies are needed to investigate whether T cell PPAR γ regulates mucosal inflammation by altering carbohydrate metabolism, colonic cell apoptosis and protein synthesis and its relation with ER stress.

CD4cre mice have significantly fewer CD4^+^FoxP3^+ ^Tregs in blood and IL10-expressing CD4^+ ^T cells in the MLN on day 7 of DSS colitis in comparison to WT mice. We have demonstrated that the accumulation of Treg in the mucosal inductive sites (i.e., MLN) is associated with prevention of chronic colitis caused by the adoptive transfer of CD4+CD45RB^hi ^T cells in SCID mice [17]. Our flow cytometry results indicate that T cell PPAR γ is required for the maintenance of IL-10-producing CD4^+ ^T cells in MLN, where intestinal immune responses are regulated, thereby inhibiting IL-6 and IL-1β expression in the gut mucosa during IBD. Specifically, the levels of IL10-expressing CD4+ T cells may be low in CD4Cre mice at the onset due to the lack of PPAR γ. Since regulatory responses are initiated in parallel with inflammatory responses, MLN Tregs are likely to increase in response to the DSS challenge, but their numbers may wane as they are recruited towards the principal site of inflammation; the colonic mucosa.

Adding to what was known about the effect of epithelial cell and macrophage-specific deletion of PPAR γ, our findings indicate that T cell PPAR γ also plays a protective role during the progression of DSS colitis, though to a lesser degree as seen in epithelial cells and macrophages. The lower impact observed with T cell PPAR γ fits with previous findings indicating that epithelial cells and macrophages are the primary inflammatory mediators during acute DSS colitis. Because T cell-mediated inflammation has been shown to be very influential in chronic stages of the disease [38,53], it may be worthwhile to assess the affect of T cell PPAR γ in chronic colitis models.

## Conclusions

Our findings indicate that T cell PPAR γ dampens the inflammatory response and tissue destruction in the later stages of experimental IBD by down-modulating expression of inflammatory cytokines and adhesion molecules. T cell PPAR γ is also important in regulating the relative abundance CD8^+ ^and CD4^+ ^T cell subsets, including Treg in the periphery and mucosal inductive sites.

## List of abbreviations used

IBD: Inflammatory bowel disease; PPAR γ: peroxisome proliferator-activated receptor γ; NF-κB: nuclear factor κB; DSS: dextran sodium sulphate; Treg: regulatory T cell; MLN: mesenteric lymph node; intgav: integrin alpha V; intgb2: integrin beta 2; ICAM-1: intracellular adhesion molecule 1; VCAM-1: vascular adhesion molecule 1; IL: interleukin; SOCS-3: suppressor of cytokine signalling 3; intgax: integrin alpha X; MAdCAM-1: mucosal addessin adhesion molecule 1.

## Competing interests

The authors declare that they have no competing interests.

## Authors' contributions

AJG analyzed data, summarized flow cytometry results, performed real-time RT-PCR and contributed to write the manuscript. SKM performed the microarray analyses and contributed to write the manuscript. WTH performed mouse studies and contributed to flow cytometry data acquisition and analysis, and performed RNA isolation. JBR and RH designed the experiments, obtained funding for this project, managed the project and wrote the manuscript.

## Pre-publication history

The pre-publication history for this paper can be accessed here:

http://www.biomedcentral.com/1471-230X/10/60/prepub

## Supplementary Material

Additional file 1 **Venn diagram showing number of genes differentially expressed on day 2 of DSS challenge**. The number inside each circle refers to number of genes differentially expressed on 2^nd ^day of DSS challenge (compared to control, i.e., day 0), for each genotype (WT or CD4cre). The number inside overlapping region of two circles refers to the number of genes that are common to both genotypes. Click here for file

Additional file 2 **Venn diagram showing number of genes differentially expressed on day 7 of DSS challenge**. The number inside each circle refers to number of genes differentially expressed on 7^th ^day of DSS challenge (compared to control, i.e., day 0), for each genotype (WT or CD4cre). The number inside overlapping region of two circles refers to the number of genes that are common to both genotypes. Click here for file

Additional file 3 **KEGG pathways modulated on day 7 of DSS challenge in CD4cre mice**. A total of 2990 genes, transcriptionally affected only on day 7 of CD4cre mice (in no other condition), were subjected to hypergeometric testing and revealed enrichment (over-representation) of four KEGG pathways: Krebs cycle, amino sugars, apopotosis and ribosome pathways. The first and last columns correspond to the KEGG identifier and name of the pathway respectively. The second and third columns (Pvalue, OddsRatio) report that there is good association between DSS challenge and these pathways. The ExpCount records the expected number of genes in the selected gene list to be found at each tested KEGG pathway, which is exceeded by the actual Count (fifth column). Sixth column (Size) corresponds to the total number of genes in that pathway. One of these pathways, 'apoptosis', is regulated by NF-κB that is repressed by PPARγ. Click here for file

Additional file 4 **Krebs (Citrate) cycle pathway from KEGG**. A total of 2990 genes, transcriptionally affected only in CD4cre mice on day 7 of dextan sodium sulfate (DSS) challenge, were subjected to hypergeometric testing for discovering the pathways significantly associated with DSS. Many genes participating in Krebs cycle were found to be down-regulated (painted green in this diagram). Click here for file

Additional file 5 **Apoptosis pathway from KEGG**. A total of 2990 genes, transcriptionally affected only in CD4cre mice on day 7 of dextan sodium sulfate (DSS) challenge, were subjected to hypergeometric testing for discovering the pathways significantly associated with DSS. Many genes participating in apoptosis were found to be up-regulated (painted red in this diagram). Click here for file

Additional file 6 **Gene Set Enrichment Analysis 7 days post DSS (Ribosome pathway)**. Panel A Gene Set Enrichment Analysis (GSEA) was performed on CD4cre and wild-type (WT) mice after 7 days of dextan sodium sulfate (DSS) challenge. The pathway "Ribosome" (KEGG Id: 03010) was found to have the lowest pathway score and significantly different from the other pathways, as observed on the Q-Q plot (not shown). Depicted here is a scatter plot in which each point corresponds to an expression signal for a gene belonging to this pathway. The line is a 45-degree diagonal. For most genes the expression values are less for CD4cre compared with WT (most points lie above the diagonal, i.e., lean toward WT), suggesting that this pathway is down-regulated in colonic mucosa of CD4cre mice on day 7 following DSS treatment. **Panel B **2990 genes, transcriptionally affected only in CD4cre mice on day 7 of dextran sodium sulfate (DSS) challenge, were subjected to hypergeometric testing for discovering the pathways significantly associated with DSS. Many genes participating in Ribosome were found to be down-regulated (painted green in this diagram). Click here for file

Additional file 7 **Supplemental Table S1 - Fold-change in gene expression after 7 days of Dextran Sodium Sulfate challenge in CD4cre mice**. The genes belong to the three KEGG pathways: Krebs (Citrate) cycle, Apoptosis and Ribosome. Click here for file
