# Supplementary material for: The Role of T cell PPAR γ in mice with experimental inflammatory bowel disease
Source: BMC Gastroenterol. 2010 Jun 10;10:60. doi: 10.1186/1471-230X-10-60 (PMC2891618; doi:10.1186/1471-230X-10-60)
Supplement: Additional file 2 — Venn diagram showing number of genes differentially expressed on day 7 of DSS challenge. The number inside each circle refers to number of genes differentially expressed on 7th day of DSS challenge (compared to control, i.e., day 0), for each genotype (WT or CD4cre). The number inside overlapping region of two circles refers to the number of genes that are common to both genotypes. [file 1471-230X-10-60-S2.DOC]

**Supplementary Figure 2**
