# Supplementary material for: The Role of T cell PPAR γ in mice with experimental inflammatory bowel disease
Source: BMC Gastroenterol. 2010 Jun 10;10:60. doi: 10.1186/1471-230X-10-60 (PMC2891618; doi:10.1186/1471-230X-10-60)
Supplement: Additional file 3 — KEGG pathways modulated on day 7 of DSS challenge in CD4cre mice. A total of 2990 genes, transcriptionally affected only on day 7 of CD4cre mice (in no other condition), were subjected to hypergeometric testing and revealed enrichment (over-representation) of four KEGG pathways: Krebs cycle, amino sugars, apopotosis and ribosome pathways. The first and last columns correspond to the KEGG identifier and name of the pathway respectively. The second and third columns (Pvalue, OddsRatio) report that there is good association between DSS challenge and these pathways. The ExpCount records the expected number of genes in the selected gene list to be found at each tested KEGG pathway, which is exceeded by the actual Count (fifth column). Sixth column (Size) corresponds to the total number of genes in that pathway. One of these pathways, 'apoptosis', is regulated by NF-κB that is repressed by PPARγ. [file 1471-230X-10-60-S3.DOC]

**Supplementary Figure 3**

| Gene to KEGG test for over-representation | | | | | | |  |
| --- | --- | --- | --- | --- | --- | --- | --- |
| **KEGGID** | **Pvalue** | **OddsRatio** | **ExpCount** | **Count** | **Size** | **Term** | |
| 00020 | 0.000 | 5.050 | 5 | 14 | 30 | Citrate cycle (TCA cycle) | |
| 04210 | 0.000 | 2.713 | 13 | 27 | 85 | Apoptosis | |
| 03010 | 0.000 | 2.666 | 13 | 27 | 86 | Ribosome | |
