# Supplementary figures and images for: The Role of T cell PPAR γ in mice with experimental inflammatory bowel disease
Source: BMC Gastroenterol. 2010 Jun 10;10:60. doi: 10.1186/1471-230X-10-60 (PMC2891618; doi:10.1186/1471-230X-10-60)

**Supplementary Figure 4**

**
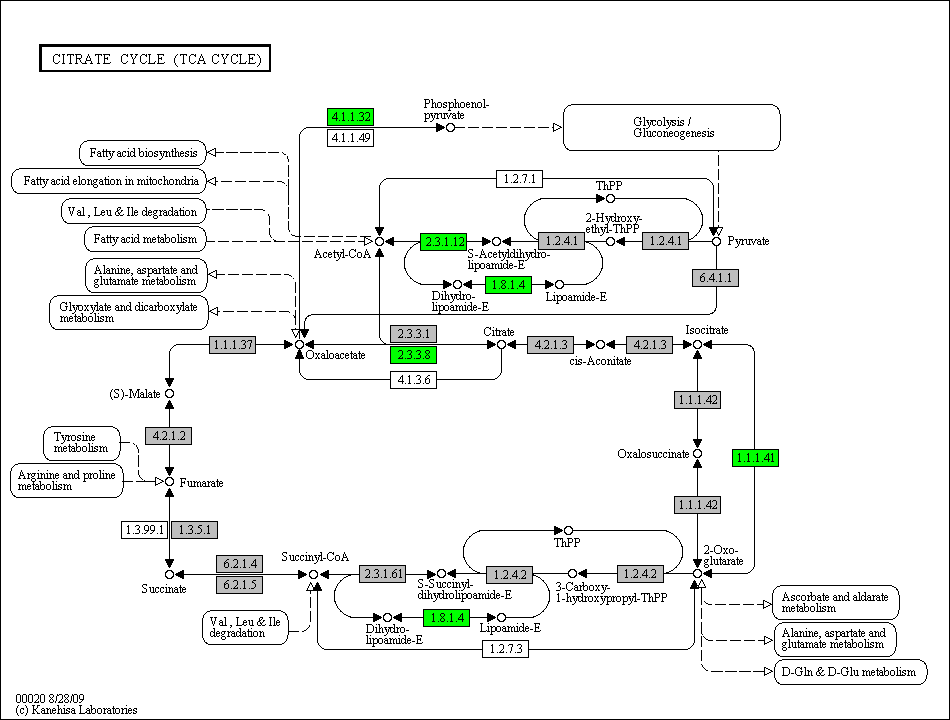
**

Supplement: Additional file 4 — Krebs (Citrate) cycle pathway from KEGG. A total of 2990 genes, transcriptionally affected only in CD4cre mice on day 7 of dextan sodium sulfate (DSS) challenge, were subjected to hypergeometric testing for discovering the pathways significantly associated with DSS. Many genes participating in Krebs cycle were found to be down-regulated (painted green in this diagram). [file 1471-230X-10-60-S4.DOC]

**Supplementary Figure 5**

**
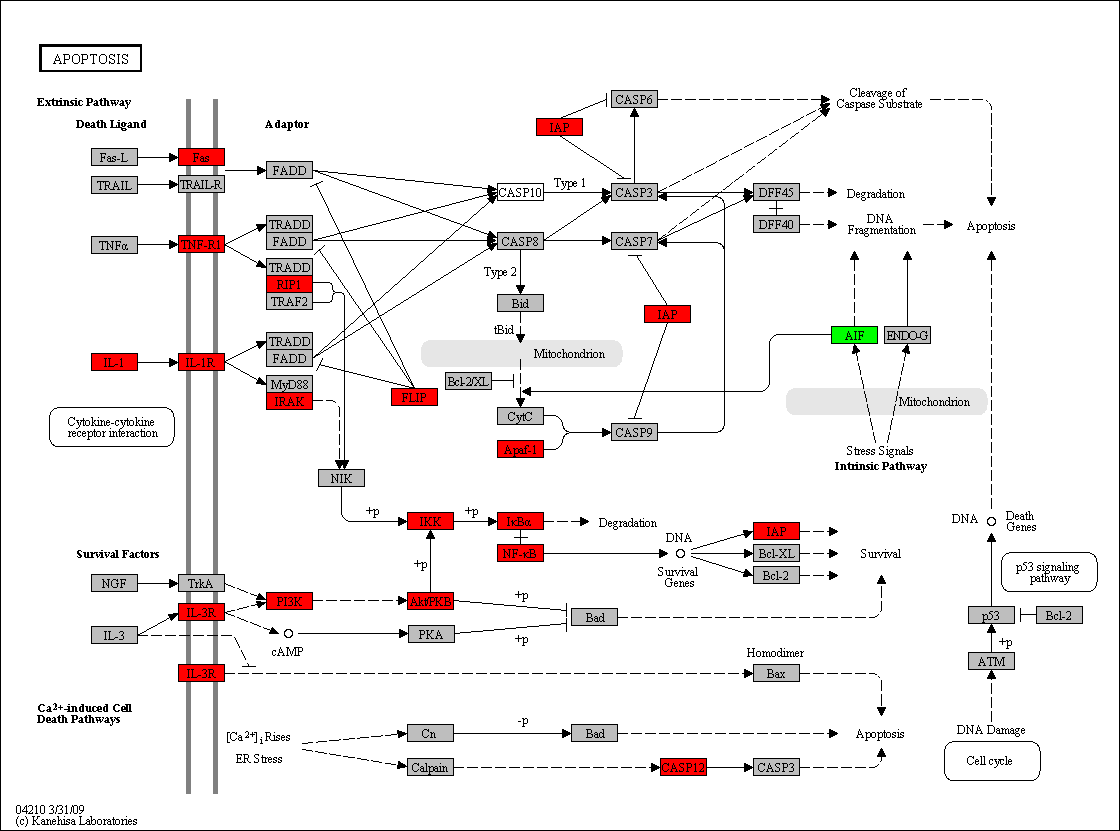
**

Supplement: Additional file 5 — Apoptosis pathway from KEGG. A total of 2990 genes, transcriptionally affected only in CD4cre mice on day 7 of dextan sodium sulfate (DSS) challenge, were subjected to hypergeometric testing for discovering the pathways significantly associated with DSS. Many genes participating in apoptosis were found to be up-regulated (painted red in this diagram). [file 1471-230X-10-60-S5.DOC]

**Supplementary Figure 6**

**Panel A**

**Panel B**

**
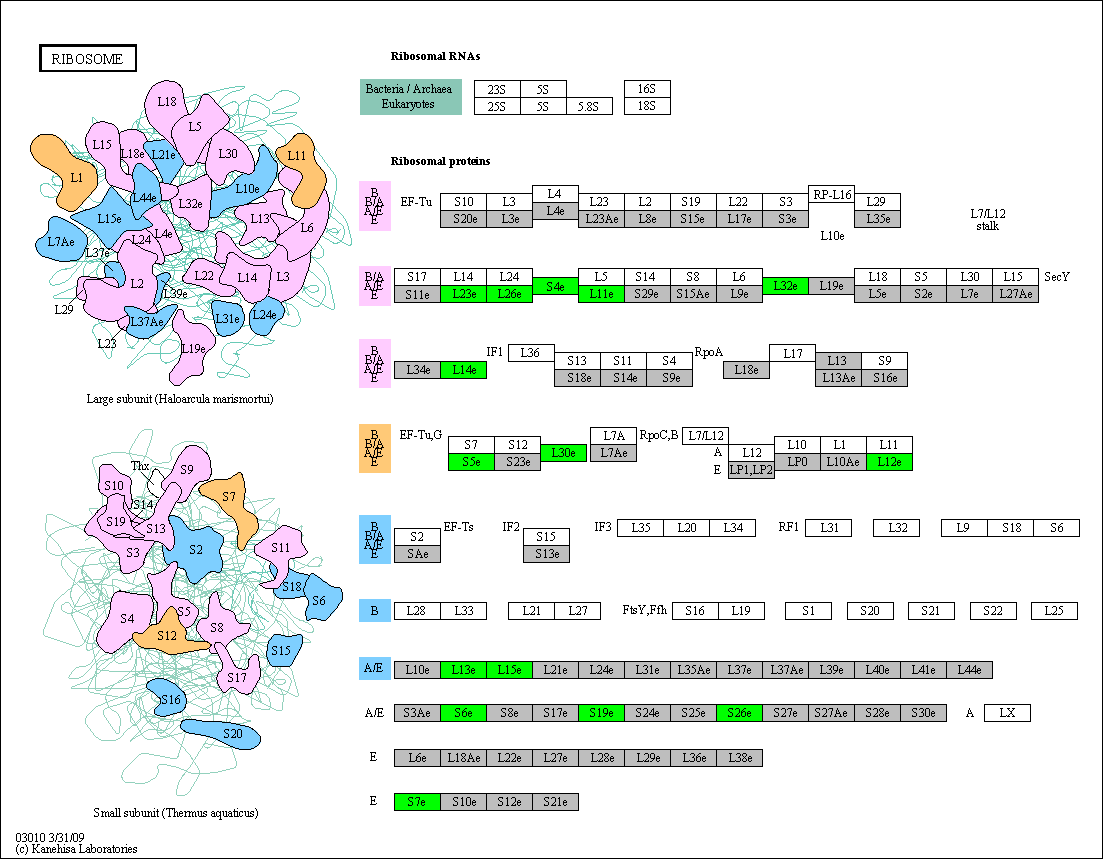
**

Supplement: Additional file 6 — Gene Set Enrichment Analysis 7 days post DSS (Ribosome pathway). Panel A Gene Set Enrichment Analysis (GSEA) was performed on CD4cre and wild-type (WT) mice after 7 days of dextan sodium sulfate (DSS) challenge. The pathway "Ribosome" (KEGG Id: 03010) was found to have the lowest pathway score and significantly different from the other pathways, as observed on the Q-Q plot (not shown). Depicted here is a scatter plot in which each point corresponds to an expression signal for a gene belonging to this pathway. The line is a 45-degree diagonal. For most genes the expression values are less for CD4cre compared with WT (most points lie above the diagonal, i.e., lean toward WT), suggesting that this pathway is down-regulated in colonic mucosa of CD4cre mice on day 7 following DSS treatment. Panel B 2990 genes, transcriptionally affected only in CD4cre mice on day 7 of dextran sodium sulfate (DSS) challenge, were subjected to hypergeometric testing for discovering the pathways significantly associated with DSS. Many genes participating in Ribosome were found to be down-regulated (painted green in this diagram). [file 1471-230X-10-60-S6.DOC]
